# The Simultaneous Treatment of PC-3 Cells with the DNA-Demethylating Agent Decitabine and S-Adenosylmethionine Leads to Synergistic Anticancer Effects

**DOI:** 10.3390/genes15121634

**Published:** 2024-12-20

**Authors:** Thomas Schmidt, Carsten Sticht

**Affiliations:** 1 Department of Anatomy and Developmental Biology, Medical Faculty Mannheim, Heidelberg University, 68167 Mannheim, Germany; 2 Department of Bioinformatics, Medical Faculty Mannheim, Heidelberg University, 68167 Mannheim, Germany

**Keywords:** decitabine, S-adenosylmethionine, PC-3 cells

## Abstract

**Background:** Epigenetic dysregulation is a common feature of cancer. Promoter demethylation of tumor-promoting genes and global DNA hypomethylation may trigger tumor progression. Epigenetic changes are unstable; thus, research has focused on detecting remedies that target epigenetic regulators. Previous studies have suggested that concordantly targeting hypomethylation and hypermethylation is beneficial for suppressing both the oncogenic and pro-metastatic functions of cancer cells. Therefore, we aimed to investigate the effect of a combination of S-adenosylmethionine (SAM) and the demethylating agent decitabine on prostate cancer cells. **Materials and Methods:** Prostate cancer cells (PC-3) were treated with SAM, decitabine, or a combination of both. Proliferation, migration, invasion, and methylation assays were also performed. A transcriptome study was conducted to detect different gene clusters between the treatment groups, followed by analyses using the Kyoto Encyclopedia of Genes and Genomes pathway and ingenuity pathway analysis. Finally, to gain information on differential gene expression, promoter methylation studies were performed. **Results:** Groups treated with decitabine, SAM, or their combination showed reduced proliferative capacity. The decitabine-treated group showed a marginal increase in cell migration and invasion, whereas the SAM-treated and combination treatment groups showed reduced invasion and migration potential. Methylation assays demonstrated the restoration of decitabine-induced demethylation in prostate cancer samples, whereas the transcriptome study revealed the upregulation of different gene clusters between the treatment groups. Methylation studies confirmed that SAM could restore the decitabine-induced demethylation of proto-oncogenes, but it did not induce the re-methylation of tumor-suppressor genes. **Conclusions:** Combination treatment with SAM and decitabine had an additive effect and did not nullify each other.

## 1. Introduction

One of the most common epigenetic hallmarks of neoplasia is alterations in DNA methylation. These alterations result in changes in chromatin structure, potentially leading to chromosomal instability, a loss of heterozygosity [1,2], and differentially expressed genes, which are essential for the development of malignancies. They are attractive targets for cancer therapy because their methylation or demethylation is reversible [3]. Over the last few decades, research has focused on developing drugs that target changes in DNA methylation patterns. The United States Food and Drug Administration has approved two inhibitors of DNA methyltransferases as cancer therapeutics for treating hematological neoplasms: azacitidine and decitabine [3]. Both drugs are incorporated into DNA strands and may irreversibly bind to DNA methyltransferases, preventing methylation of the daughter strand during cell division [3]. In addition, the application of DNA-demethylating agents may induce viral mimicry triggered by the upregulation of the expression of endogenous retroviral sequences, leading to an immune response against tumor cells [4]. However, they have been used cautiously owing to their side effects, resistance formation, and limited success [3]. However, the development of remedies dealing with cancer-induced demethylation and hypomethylation of the genome remains difficult, mostly because of unknown mechanisms, although the loss of DNA methylation was discovered in the early 1980s [5]. However, the in vitro application of the principal methyl donor, S-adenosylmethionine (SAM), has demonstrated anti-proliferative and anti-metastatic effects in cancer cell lines [6,7]. SAM-mediated effects on histone and promoter methylation and miRNA expression in cancer cell lines have been confirmed [6,7,8]. Currently, SAM, which is classified as a nutraceutical agent in the US, is approved for the treatment of illnesses, such as osteoarthritis, fibromyalgia, and depression, with almost no side effects [9]. However, it has not yet been approved for use in cancer treatments. This may be due to poor bioavailability resulting from substantial first-pass effects and rapid hepatic metabolism [10,11]. Under physiological conditions, the half-life of SAM is 2 h [10]. Nevertheless, the impact of SAMs on enzymes involved in nucleic acid methylation has been proven [12,13]. SAM may induce both methylation and demethylation based on different effects on the enzymes involved, owing to differences in its concentration [6,7].

Generally, methylating and demethylating agents lack specificity for the promoters of tumor-suppressor genes or proto-oncogenes, respectively. However, this may have adverse consequences [14,15]. Thus, we hypothesized that the simultaneous application of DNA-demethylating agents and SAM could reverse these effects, as recently observed in breast cancer cells [14,15]. Therefore, we aimed to study the effects of the simultaneous treatment of prostate cancer cells with a combination of decitabine and SAM.

## 2. Material and Methods

### 2.1. Cell Culture and Treatment

Prostate cancer cells (PC-3) were purchased from the Leibniz Institute (Braunschweig, Germany). They were grown in an RPMI 1640 medium (supplemented with 10% fetal calf serums (FCS) and 1.2% penicillin/streptomycin (Gibco GmbH, Schwerte, Germany)) in an incubator at 37 °C. The PC-3 cells were treated with a vehicle (comprising 5 mM sulfuric acid and 10% ethanol in PBS), 200 µM of SAM (New England Biolabs, Ipswich, MA, USA), 1 µM of decitabine (Biomol, Hamburg, Germany), or a combination of both (200 µM of SAM and 1 µM of decitabine) for 24, 72, and 120 h. Under all conditions, we changed the medium every other day.

### 2.2. Proliferation Assay

We used the CellTiter 96^®^ AQ_ueous_ One Solution Cell Proliferation Assay (Promega, Walldorf, Germany) as previously described [6]. Briefly, PC-3 cells were grown in 96-well plates (2000 cells/well) and treated with 200 µM of SAM or the vehicle. We measured the cell viability after 24, 72, and 120 h (The most effective period was 120 h) of incubation. Three independent experiments were performed. Data are expressed as percentages of control [6].

### 2.3. Invasion and Migration Assay

In vitro colorimetric Millipore cell invasion/transendothelial migration assay kits (Sigma-Aldrich, Taufkirchen, Germany) were used according to the manufacturer’s instructions. PC-3 cells were pretreated with 200 μM of SAM, 1 µM of decitabine, a combination of both, or PBS for 24, 72, or 120 h. Subsequently, 50,000 cells were seeded into 8.0 µm hanging PET inserts (PTEP12H48) for the migration assay. The in vitro invasive ability of PC-3 cells was determined using the QCM ECMatrix Cell Invasion Assay Kit (ECM550) (Sigma-Aldrich, Taufkirchen, Germany). The cells were measured as described above. Cells (50,000) were plated in a medium containing 10% FCS in the inserts. The medium in the culture plates contained 20% FCS to create a gradient. For both assays, the cells were incubated for 24 h. After detachment and lysation, the cells were stained with a colorimetric dye. The intensity of the colorimetric signal was used to assess the number of invading or migrating cells. The colorimetric signal was read with a plate reader using a 560 nm filter. Invasion and migration rates were expressed as percentages relative to the controls.

### 2.4. Quantification of Global DNA Methylation

Global DNA methylation was measured using a colorimetric 5-Methyl Cytosine Assay Kit (ab117128; Abcam, Cambridge, UK) as previously described [4]. The DNA was extracted from treated and untreated PC3 cells (treated for 120 h) using the *Quick*-DNA/RNA Miniprep Kit (Zymo Research, Freiburg, Germany). After the DNA quality was checked, the quantity of the DNA was measured using a NanoDrop spectrophotometer (Thermo Fisher Scientific, Waltham, MA, USA). An amount of 100 ng of DNA was used per reaction to assess the percentage of the methylated DNA. Absorbance was measured using a Tecan microplate reader (Infinite^®^ F50, Tecan, Switzerland) at 450 nm after incubation for 10 min.

### 2.5. RNA Sequencing (RNA-seq)

For RNA-seq of the control and treated PC-3 samples, total RNA was isolated after treatment using the RNeasy Universal Mini Kit. RNA quality was measured on a Bioanalyzer 2100 using an RNA 6000 Nano Kit (Agilent, Waldbronn, Germany). After standard library preparation, sequencing was performed using the BGISEQ-500 platform. RNA-seq datasets were deposited in the Gene Expression Omnibus under the accession numbers GSE71070 and GSE266129.

### 2.6. Bioinformatic Analysis

Using R (version 3.6.3) and Bioconductor (version 3.9) in RStudio (version 1.1.463), RNA-seq raw data were filtered. Subsequently, the quality of the clean sequencing reads was controlled by FastQC (Babraham Bioinformatics), and low-quality reads were removed using TrimGalore (version 0.6.4). The filtered reads were subjected to alignment to the human genome GRCh38.p13. The reads were counted using kallisto version 0.46.1 [16]. The voom function in the limma package (Version 3.46.0) was used to transform count data into log2-counts per million [17]. The limma package was also used to carry out a differential expression analysis. A false positive rate of α = 0.05, with false discovery rate (FDR) correction, was established as significant. Heatmaps and clustering were created using the ggplot2 package (version 2.2.1)/complex heatmap package (version 2.0.0). Enrichment analysis was performed using the fgsea, enrichment browser, and Enrichr packages (Version 3.2). Pathway analysis was performed using the fgsea package (Version 3.2) [18] and the enrichment browser package (Version 3.2) [19] in R, using pathway information from the Kyoto Encyclopedia of Genes and Genomes (KEGG) database (https://www.genome.jp/kegg/pathway.html (accessed on 15 April 2024)).

### 2.7. Ingenuity Pathway Analysis (IPA) of Upstream Regulators

We used the commercial QIAGEN IPA (QIAGEN, Hilden, Germany; www.qiagen.com/ingenuity, accessed on 31 October 2024) software to perform an in silico analysis of the upstream regulators and top diseases in which the differentially expressed genes of the obtained datasets were involved. The Bonferroni method was used to correct the detection *p*-value, and the significance threshold was set at 0.05.

### 2.8. Promoter Methylation Analysis of Putative Proto-Oncogenes and Tumor-Suppressor Genes

To quantify DNA methylation in the promoters, we used the OneStep PLUS qMethyl PCR Kit (Zymo Research, Freiburg, Germany), according to the manufacturer’s instructions. Each analyzed sample contained 20 ng of genomic DNA in 5 µL of RNase/DNase-free water. We set up a test reaction and reference reaction mixtures for each sample and each standard (human methylated and unmethylated DNA), each of which was analyzed in parallel with both the test and reference reaction mixtures. The test reaction mixture contained methyl-specific restriction enzymes (MSREs) that selectively digested unmethylated DNA while leaving the methylated DNA intact. Subsequently, only the methylated DNA was amplified in the test reaction. The reference reaction mixture did not contain MSREs; therefore, both methylated and unmethylated DNA were amplified. To measure CpG methylation in promoter regions of *IFIT5* (Interferon induced protein with tetratricopeptide repeats 5) *STC2* (Stanniocalcin 2), and *TP63* (tumor protein p63), putative promoter sequences and CpG islands upstream of the transcription start sites were predicted using the free accessible websites “DataBase of CpG Islands and Analytical Tools” (http://dbcat.cgm.ntu.edu.tw/ (accessed on 15 November 2024)) and Methprimer 2.0 (http://www.urogene.org/methprimer2/ (accessed on 15 Novemver 2024)). The sequences (Supplementary Figure S1) contained at least three MSRE sites for accurate methylation assessment. Primer3 software (https://primer3.ut.ee (accessed on 16 November 2024)) was used to design primers listed in Table 1. The accuracy of the sequences and primers was verified using the NCBI BLASTN tool. qMethyl PCR was performed using a LightCycler 480 (Roche, Basel, Switzerland). The conditions were as follows: 30 °C for 12 h (digestion), 95 °C for 8 min, 97 °C for 2 min, and 40 cycles of 97 °C for 20 s and 58 °C for 1 min. The following equation was used to calculate the percentage of DNA methylation at the selected genomic region: Percent Methylation = 100 × 2^−ΔCt^ (ΔCt = the Ct value from the test reaction minus the Ct values from the reference reaction).

## 3. Results

### 3.1. The Combination of SAM and Decitabine Synergizes the Suppression of Growth, Migration, and Invasion in PC-3 Cells

**Figure 1:**
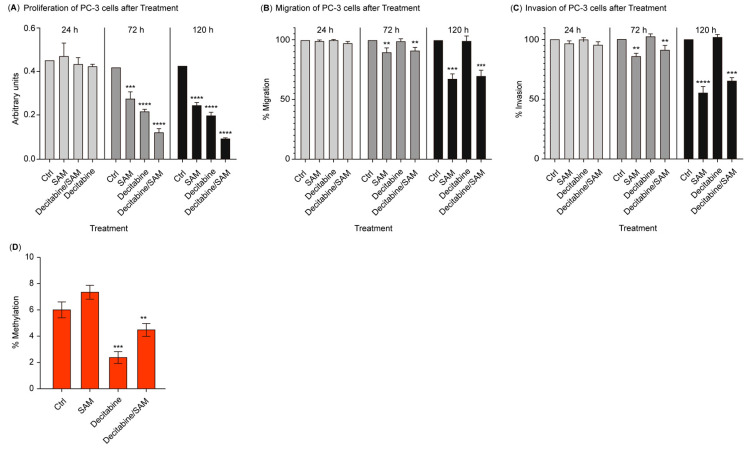
Effects of SAM, decitabine, and the combination of both on cell proliferation (**A**); migration (**B**); and invasion (**C**) in vitro. PC-3 cells were treated with the vehicle only (control), SAM (200 μM), decitabine (1 μM), and a combination of SAM and decitabine (200 µM of SAM and 1 µM of decitabine) every day for 24, 72, and 120 h. The percentage changes in cell proliferation, migration, and invasion relative to the control groups at different time points are shown as bar graphs. The results are presented as mean ± SEM. (**D**) SAM reverses decitabine-induced global demethylation. Decitabine alone significantly reduced the 5-mC methylation level in PC-3 cells. Global demethylation could be inhibited by combining the decitabine treatment with SAM. Statistical analysis was conducted using ANOVA followed by Student’s *t*-test, and significant differences are indicated by asterisks ** *p* < 0.001; *** *p* < 0.0001; and **** *p* < 0.00001). SAM, S-adenosylmethionine.

To assess the combined effect of SAM and decitabine on prostate cancer cells, PC-3 cells were treated with 200 µM of SAM, 1 µM of decitabine, or a combination of both. They compared the proliferation, migration, and invasion potential of the treated cells to the untreated controls. Decreased proliferation rates were detected in treated PC-3 cells in a time-dependent manner. After 24 h, no significant differences were observed between the groups (Figure 1A). After 72 h, the SAM- and decitabine-treated groups showed a decrease in proliferation of approximately 65% and 55% of the levels detected in the controls (Figure 1A). The combination of both treatments reduced 35% of the levels found in the control samples. After 120 h, the maximum decrease in proliferation was observed, with a reduction in the SAM- and decitabine-treated groups to 62% and 54% of the controls and, regarding the combination of both, to 22% of the controls (Figure 1A). Decitabine treatment increases the migration and invasion potential of cancer cells; therefore, to assess whether combination treatment with decitabine and SAM could restore this effect, we performed invasion and migration assays using SAM and decitabine-treated PC-3 cells. After 24 h, no significant differences were detected between the groups and their respective controls (Figure 1B,C). After 72 h, SAM and decitabine/SAM-treated groups showed a slightly significant reduction in migration (89/91% of controls) and invasion potential (87/90% of controls) (Figure 1B,C). The decitabine-treated group showed a moderate increase in migration (102% of controls) and invasion (101% of controls). After 120 h, all three treatment groups exhibited similar, even more pronounced effects. The migration and invasion of the SAM and decitabine/SAM groups showed a significant decrease when compared with those of their respective controls (SAM: migratio*n* = 68% of the control, invasion = 62% of the control; SAM/decitabine: migration = 73% of the control, invasion = 66% of the control). The decitabine group displayed moderate increases in migration (103% of the control) and invasion (105% of the control) (Figure 1B,C).

### 3.2. Treatment with SAM Prevents Global Hypomethylation Induced by Decitabine

To test whether treatment with SAM leads to the decitabine-induced inhibition of demethylation of the cancer genome, we investigated the DNA of treated PC-3 cells and controls using a colorimetric 5-Methyl Cytosine Assay Kit. SAM alone did not further increase the global state of methylation of PC-3 cells (Figure 1D); however, decitabine treatment caused significant global demethylation of the cancer genome (Figure 1D). Nevertheless, SAM treatment in combination with decitabine treatment resulted in the significant inhibition of global demethylation triggered by decitabine alone (Figure 1D).

### 3.3. The Combination Treatment of SAM and Decitabine Affects the Transcriptome of PC-3 Cells

**Figure 2:**
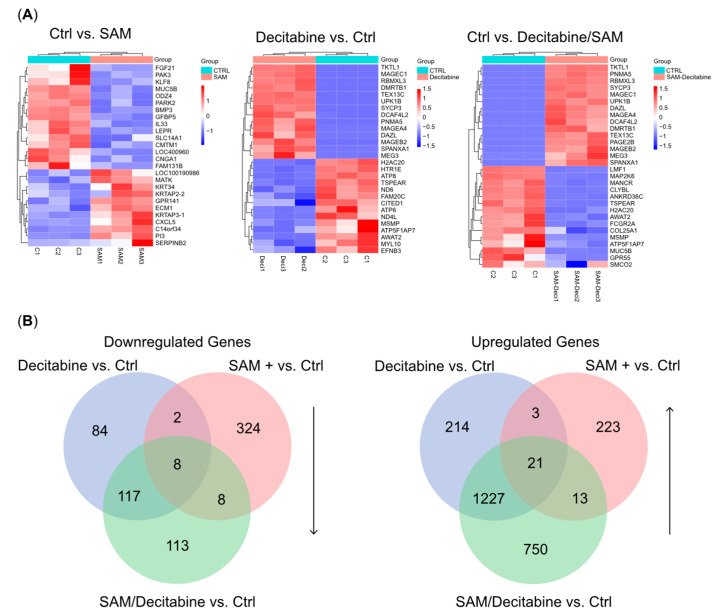
Transcriptome analyses of PC-3 cells treated with SAM, decitabine, or a combination of both. (**A**) Heatmaps of differentially expressed genes (DEGs) obtained from transcriptome studies of each treatment group in relation to the controls (*n* = 3/group); and (**B**) Venn diagrams showing the overlap of upregulated and downregulated DEGs among the different groups. SAM, S-adenosylmethionine.

**Figure 3:**
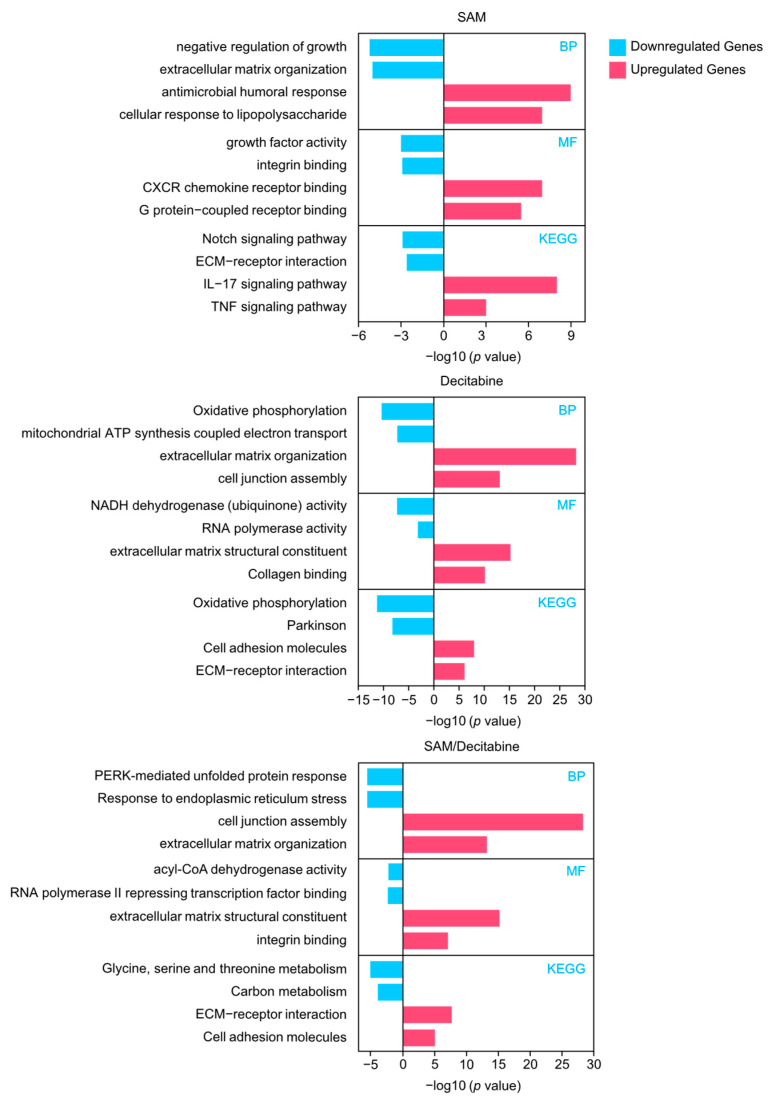
Functional enrichment analysis of the up- and downregulated genes in different treatment regimens, SAM treatment alone, decitabine treatment alone, and a combination treatment with decitabine/SAM. Abbreviations: BP, biological process; MF, molecular function; SAM, S-adenosylmethionine.

We performed a transcriptome study to evaluate differentially expressed genes mediated by the distinct treatments of samples (*n* = 3) compared with controls. Our data revealed significant changes in gene expression in the groups treated with single agents (decitabine and SAM), in contrast to that with the combination treatment. Single-agent treatment with decitabine caused significant alterations in the expression of 1679 genes (211 downregulated and 1468 upregulated). In contrast, single-agent treatment with SAM caused changes in the expression of 605 genes (342 downregulated and 263 upregulated) (Figure 2A,B). By creating Venn diagrams for the upregulated and downregulated genes, we detected 863 genes (113 downregulated and 750 upregulated) that were significantly affected exclusively by the combination treatment with decitabine and SAM but not by single treatment with decitabine or SAM (Figure 2B). Among these genes, *ANXA2R* (Annexin A2 Receptor), *RBM3* (RNA-binding Protein 3), and *SUCLG2* (Succinyl-CoA ligase [GDP-forming] subunit β), which may act as cancer drivers, were downregulated. In contrast, we detected *ST3GAL5* (Lactosylceramide α-2,3-sialyltransferase), *ITGA7* (Integrin α 7), and *RPRM* (TP53-dependent G2 arrest mediator homolog), which may act as tumor-suppressors (Supplementary Tables S1–S6). To gain more insight into the biological functions of the different treatment regimens, we performed GO-term enrichment and KEGG pathway analyses between differentially expressed genes in PC-3 cells treated with SAM, decitabine, or a combination of both (Figure 3; Supplementary Tables S1–S6). In cells treated with the combination therapy, we detected the enrichment of upregulated genes involved in pathways (KEGG), e.g., “ECM–receptor interaction” and “cell adhesion molecules”; biological processes (BPs), e.g., “cell junction assembly” and “extracellular matrix organization”; and molecular functions (MFs), e.g., “integrin binding”. These are all crucial processes for the integrity of epithelia and the extracellular matrix. Downregulated genes were enriched in processes involved, for example, in the “regulation of response to endoplasmic reticulum stress” (BP) and the general regulation of “carbon metabolism” (KEGG).

### 3.4. IPA Identifies Three Prime Repair Exonuclease (TREX)1, Stimulators of Interferon Genes (STING)1, and TP63 as Upstream Regulators in PC-3 Cells Treated with Combination Therapy (SAM/Decitabine)

**Figure 4:**
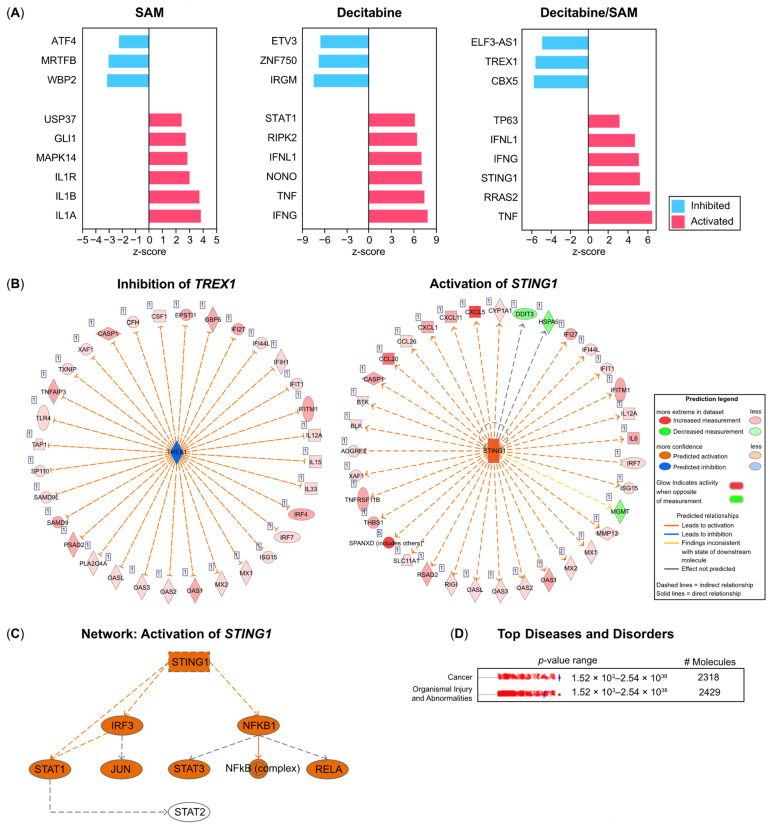
Upstream transcription regulator analysis. (**A**) A list of predicted significantly activated (red bars) and inhibited (blue bars) upstream regulators in each treatment group depicted as bar graphs. A z-score greater than 2.0 indicates significant activation, and a z-score less than 2.0 indicates inhibition; (**B**) TREX1 (inhibited) and STING1 (activated) are among the upstream regulators predicted to be inhibited by SAM and decitabine treatment. The target molecules of the two factors are shown (for further details, see the Prediction legend); (**C**) Mechanistic network analyses of STING1 show the network of targets that are possibly affected by the combination treatment; and (**D**) the top three diseases and disorders enriched by the IPA “Core Analysis” for the genes differentially expressed between the controls and samples treated with the combination of SAM and decitabine. SAM, S-adenosylmethionine.

To detect potential upstream regulators mediating the differential expression of genes in the obtained datasets, we performed IPA. IPA can identify any gene or chemical that has a proven effect on the expression of gene clusters. In each of the three groups (SAM, decitabine, and SAM/decitabine), we examined the most significant upstream regulators that were either activated (*n* = 6) or inhibited (*n* = 3) (Figure 4A, Supplementary Table S7). For the combination treatment (SAM/decitabine) (Figure 4A, Supplementary Table S7), we found clusters of enriched differentially expressed genes (DEGs) that may be regulated by activated *TNF* (Tumor necrosis factor), *TP63*, and *STING1* or inhibited *TREX1* (Figure 4A, Supplementary Table S7). *STING1* and *TREX1* have been previously described in conjunction with upregulated interferon signaling and the promotion of antitumor immunity in cancer cells. Differentially expressed downstream target genes of the two factors are shown in Figure 4B (Supplementary Table S7). The IPA mechanistic network analysis tool for *STING1* predicted interconnections with *IRF3* (Interferon regulating factor 3), which is essential for the transcriptional activation of interferons, and *STAT1*, which is involved in the upregulation of interferon-stimulated genes (Figure 4C). Additionally, activated *STING1* was predicted to affect the activity of *NFKB1* (nuclear factor kappa B subunit 1) (Figure 4C), which may mediate early immune responses, such as inflammation. Overall, these data point to the activation of the innate immune system as a reaction to combination treatment with SAM/decitabine. Finally, predictions related to the differential expression patterns placed cancer and organismal injury and abnormalities among the top three diseases and disorders (Figure 4D).

### 3.5. SAM Inhibits the Decitabine-Driven Hypomethylation in Proto-Oncogenes But Does Not Inhibit the Hypomethylation in Tumor-Suppressor Genes

**Figure 5:**
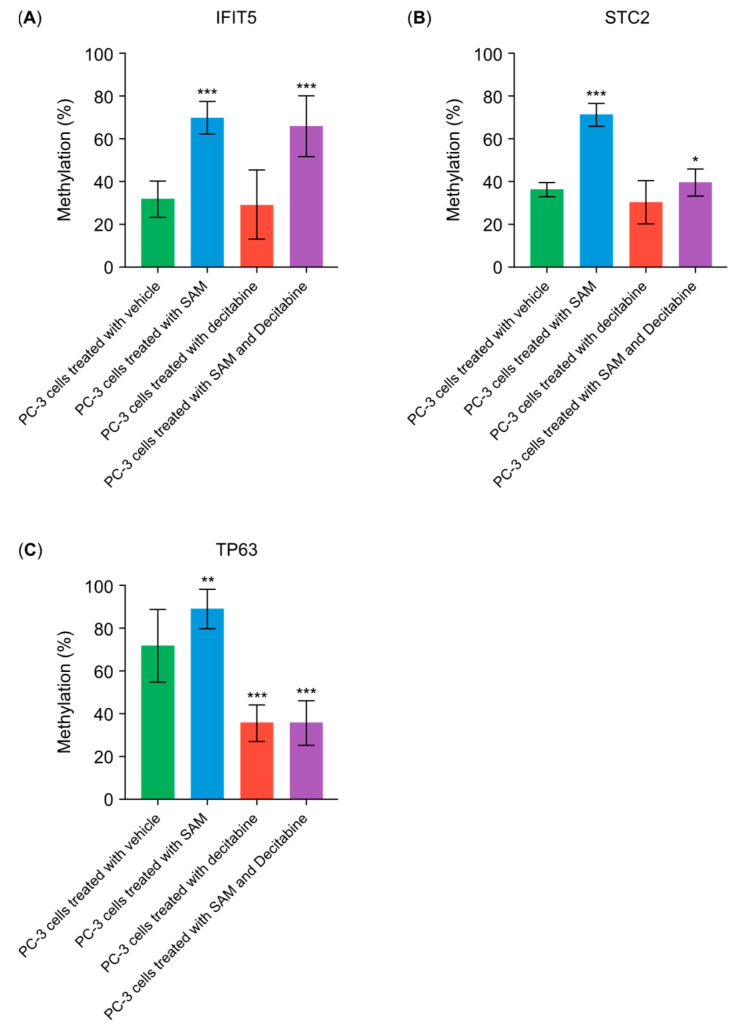
Promoter methylation level for selected genes. SAM reverses the hypomethylation of putative proto-oncogenes (**A**) *IFIT5* and (**B**) *STC2* triggered by decitabine treatment. DNA isolated from PC-3 cells treated with either 200 μM of SAM and/or 1 μM of decitabine for 120 h was subjected to OneStep PLUS qMethyl PCR. The graphs represent the percentage of methylation of the examined DNA sequences; and (**C**) SAM does not inhibit the decitabine-driven hypomethylation of the putative tumor-suppressor gene *TP63*. Graphs represent the percentage of methylation of the examined DNA sequences. Statistical analysis was conducted using ANOVA followed by Student’s *t*-test, and significant differences are indicated by asterisks (* *p* < 0.01; ** *p* < 0.001; *** *p* < 0.0001). SAM, S-adenosylmethionine.

To test whether the actions of SAM involve inhibiting the decitabine-induced promoter hypomethylation of proto-oncogenes, we investigated DNA methylation upstream of two genes known to promote carcinogenesis. *IFIT5* induces epithelial–mesenchymal transition (EMT) in prostate cancer cells. Untreated PC-3 cells showed a methylation level of 36% in the examined DNA sequences. SAM treatment increased the methylation levels (70%), whereas treatment with decitabine alone resulted in a reduction (30%) (Figure 5A). The combination treatment almost restored the SAM-induced hypermethylation of the examined DNA sequences (67%). The analysis of sequences in the 5ʹ upstream region of *STC2*, a gene known to promote prostate cancer proliferation, revealed that the combination treatment induced at least a slight increase in DNA methylation (40%) compared with the controls (35%) (Figure 5A). To test if decitabine, in turn, could re-establish the promoter hypomethylation of tumor-suppressor genes, we investigated the 5ʹ upstream region of *TP63*, which may be essential for the integrity of the prostate basal epithelium. Untreated PC-3 cells displayed a methylation level of 73%, which increased to almost 90% in SAM-treated PC-3 cells (Figure 5B). Treatment with decitabine alone decreased methylation levels to 37% (Figure 5B). The combination treatment showed no significant increase in methylation levels (40%) compared to decitabine treatment alone. This indicated that the promoter sequence of *TP63* remained at a low methylation level, even upon the addition of SAM (Figure 5B).

## 4. Discussion

In this study, we examined the effects of a combination treatment consisting of SAM (a methylating agent) and decitabine (a demethylating agent) on prostate cancer cells. Demethylating agents, such as decitabine, have the potential to reduce the proliferation of hematological and solid malignancies in vitro and in vivo [20]. However, whether a single application of demethylating agents could promote metastasis via the promoter demethylation of proto-oncogenes remains unclear [14,15]. Several studies have suggested that combination treatment with SAM, known to decrease the migration and invasion potential of cancer cell lines [14,15], could restore the promoter methylation of proto-oncogenes. Therefore, the effects of these two drugs can be combined. SAM may reverse the demethylating effect of decitabine on the promoters of tumor-suppressor genes, thus eliminating the anti-carcinogenic effects [14]. In this study, we detected decreased proliferation and diminished invasion and migration potential in prostate cancer cells treated with combination therapy. In contrast, a single treatment with decitabine caused the downregulation of proliferation but a marginal increase in migration and invasion potential. These results support the hypothesis that both agents have synergistic effects on the proliferation, migration, and invasion of prostate cancer cells. To corroborate these assumptions and confirm that SAM may inhibit decitabine-driven metastasis, we performed a global DNA methylation assay, which revealed the inhibition of decitabine-induced DNA demethylation upon SAM treatment. To examine the molecular mechanisms underlying the improved anti-carcinogenic effects, we performed transcriptome analyses on prostate cancer cells (PC-3) following treatment with decitabine, SAM, or a combination of both. Different gene clusters were identified in the treatment groups. A total of eight downregulated and 24 upregulated genes were commonly expressed in all three groups. In general, we found more similarities in gene expression between the combination treatment and decitabine treatment and GO-term enrichment/KEGG pathway analysis, reflecting the upregulation of extracellular matrix organization and cell–cell contacts. However, 863 DEGs (113 downregulated and 750 upregulated) were exclusively detected in the combination treatment group. Among these genes, we found examples of proto-oncogenes that were downregulated, such as succinate-CoA ligase GDP-forming β subunit (*SUCLG2*), which is known to drive neuroendocrine differentiation of prostate cancer after androgen deprivation therapy [21]. However, many upregulated tumor-suppressor genes have been discovered, for example, integrin alpha7 (*ITGA7*), which is known to induce apoptosis in prostate cancer cells in vitro and in vivo [22] by interacting with the high-temperature requirement A2 (*HtrA2*), which mediates cell death in cancer cells [22]. Overall, the combination treatment represents not only a summation of both single treatments but also creates transcriptomic signatures.

These results prompted us to investigate the molecular differences between the datasets obtained using RNA-seq. We performed a QIAGEN IPA. IPA provides a web-based analytical tool for omics data, making it easy to identify the most significant signaling or metabolic pathways, molecular networks, and biological functions in a list of genes. Focusing on transcription regulators that mediate the differential expression of gene clusters discovered by RNA-seq, *TREX1* was identified as an upstream regulator and predicted to be inhibited. *TREX1* represents a 3–5′ DNA exonuclease, acting as a DNA-degrading enzyme. This enzyme clears cytosolic DNA [23,24] and usually activates pattern recognition receptors (PRRs). PRRs, which are considered a part of the innate immune system, are known to detect nucleic acids within cytosolic and endosomal compartments [23,24]. Subsequently, PRR binding to nucleic acids initiates signaling cascades, releasing inflammatory molecules, such as type I interferons [23,24]. This boosts the recruitment of immune cells that can detect and counteract cancer cells. As PRRs do not sufficiently differentiate between exogenous and endogenous nucleic acids, *TREX1* deficiency may trigger chronic inflammatory and autoimmune diseases [23,24]. Various conditions, apart from infectious (viral) diseases, lead to increased levels of cytosolic single- and/or double-stranded DNA. In cancer cells, the most important mechanism is the formation of micronuclei due to the failure of chromosome segregation during mitosis [25]. The downregulation of *TREX1* may activate cyclic GMP-AMP synthase (cGAS)/STING-dependent nucleic acid sensing [26]. cGAS represents one of the above-mentioned PRRs. Its contact with nucleic acids initiates the synthesis of cyclic GMP-AMP, which subsequently binds to *STING*. *STING* is usually downregulated in cancer cells; however, it was predicted to function as an activated upstream regulator in our study. Under normal conditions, it leads to the phosphorylation/activation of transcription factors, such as IRF3 and NF-κB, promoting the transcription of interferon genes or even initiating apoptosis [27]. Overall, identifying inhibited *TREX1* and activated *STING* as upstream regulators in our datasets suggests a molecular network. If functional, they can prevent cancer cells from escaping immune cell detection and trigger programmed cell death [27] upon combination therapy. IPA core analysis further identified *TP63* as an upstream regulator. *TP63* encodes p63, which shares structural similarity with p53. p63, in turn, is known to activate several p53 target genes, mediating anti-proliferative effects and thus acting as a tumor-suppressor in some cancers [28]. *TP63* is essential for basal epithelial cell development in prostate p63-positive basal cells, including prostate epithelial stem cells, which are required for proper prostate development [28]. Luminal epithelial cells (secretory active cells) do not express p63, and it was assumed that differentiation into secretory active cells depends on the downregulation of p63 [28]. Consequently, prostate adenocarcinoma cells mostly lack p63 [29]. p63 has two promoters and encodes two different proteins: those containing (TAp63) and lacking (ΔNp63) [29], an N-terminal transactivating domain homologous to that present in p53 [29]. In the prostate cancer cell line, we used PC-3, where only a low expression of both p63 isoforms was found to be optimal [29]. The upregulation of both isoforms leads to the overexpression of *miR-205*, which is essential for EMT inhibition [29]. In the combination and single-decitabine groups, *TP63* expression was upregulated in the transcriptome, whereas in the single-SAM group, it was significantly downregulated. To determine the mechanism underlying differential expression, we performed promoter methylation analysis of *TP63*. We observed high methylation levels in the control and SAM-treated samples. The methylation percentage was much lower in the decitabine group and the combination group. Therefore, the decitabine-induced effects on the promoter of this tumor-suppressor gene cannot be nullified by SAM treatment. Additionally, promoter methylation analyses of *IFIT5* and *STC2*, two proven proto-oncogenes [30,31], showed that the application of decitabine did not interfere with the SAM-induced silencing of genes mediated by promoter methylation. Thus, we showed that decitabine- and SAM-driven effects were not antagonistic. However, we did not perform a whole-genome methylation analysis, which is a limitation of our study. Therefore, the observed methylation effects representing individual events cannot be ruled out. Another limitation of our study is the lack of in-depth experimentation to evaluate the hypothesis raised by IPA.

In conclusion, combination treatment with SAM and decitabine increased the effects of the two agents. Moreover, this treatment is likely to induce an anticancer immune response.

## Figures and Tables

**Table 1:** Primer sequences for qMethyl PCR.

Primer for Methyl-qPCR	Sequence (5′–3′)
*STC fw*	CCGACTCAGGAGAGCTC (Tm = 56 °C)
*STC rev*	CCCAGCCGTGTCACATG (Tm = 56 °C)
*IFIT5 fw*	GAAGCAGGGACTTAAGTTTC (Tm = 58 °C)
*IFIT5 rev*	GCTCTTGAGCTCTTCTATTAA (Tm = 58 °C)
*TP63*	ACAACAGTAGAGAGGATGCC (Tm = 57 °C)
*TP63*	CTCAAACTTACACTGTATTGA (Tm = 56 °C)

## Data Availability

The original contributions presented in the study are included in the article/Supplementary Materials, further inquiries can be directed to the corresponding author.

## Supplementary Materials

The following supporting information can be downloaded at https://www.mdpi.com/article/10.3390/genes15121634/s1, Supplementary Figure S1 Predicted promoter sequences for *STC2*, *IFIT5*, and *TP63* used for methylation analysis. Supplementary Table S1 GO-term enrichment and KEGG pathway analysis of downregulated transcripts in PC-3 cells treated with SAM. Supplementary Table S2 GO-term enrichment and KEGG pathway analysis of upregulated transcripts in PC-3 cells treated with SAM. Supplementary Table S3 GO-term enrichment and KEGG pathway analysis of downregulated transcripts in PC-3 cells treated with decitabine. Supplementary Table S4 GO-term enrichment and KEGG pathway analysis of upregulated transcripts in PC-3 cells treated with decitabine. Supplementary Table S5 GO-term enrichment and KEGG pathway analysis of downregulated transcripts in PC-3 cells treated with SAM and decitabine. Supplementary Table S6 GO-term enrichment and KEGG pathway analysis for upregulated transcripts in PC-3 cells treated with SAM and decitabine. Supplementary Table S7 Ingenuity pathway analysis of upstream regulators for PC-3 cells treated with decitabine and SAM.

## References

[1] Ilango S., Paital B., Jayachandran P., Padma P.R., Nirmaladevi R. (2020). Epigenetic alterations in cancer. Front. Biosci..

[2] Kanai Y., Hirohashi S. (2007). Alterations of DNA methylation associated with abnormalities of DNA methyltransferases in human cancers during transition from a precancerous to a malignant state. Carcinogenesis.

[3] Jung I., An J., Ko M. (2023). Epigenetic Regulators of DNA Cytosine Modification: Promising Targets for Cancer Therapy. Biomedicines.

[4] Roulois D., Loo Y.H., Singhania R., Wang Y., Danesh A., Shen S.Y., Han H., Liang G., Jones P.A., Pugh T.J. (2015). DNA-Demethylating Agents Target Colorectal Cancer Cells by Inducing Viral Mimicry by Endogenous Transcripts. Cell.

[5] Gama-Sosa M.A., Slagel V.A., Trewyn R.W., Oxenhandler R., Kuo K.C., Gehrke C.W., Ehrlich M. (1983). The 5-methylcytosine content of DNA from human tumors. Nucleic Acids Res..

[6] Schmidt T., Leha A., Salinas-Riester G. (2016). Treatment of prostate cancer cells with S-adenosylmethionine leads to genome-wide alterations in transcription profiles. Gene.

[7] Mathes A., Duman M.B., Neumann A., Dobreva G., Schmidt T. (2024). S-adenosylmethionine treatment affects histone methylation in prostate cancer cells. Gene.

[8] Shukeir N., Pakneshan P., Chen G., Szyf M., Rabbani S.A. (2006). Alteration of the methylation status of tumor-promoting genes decreases prostate cancer cell invasiveness and tumorigenesis in vitro and in vivo. Cancer Res..

[9] Fetrow C.W., Avila J.R. (2001). Efficacy of the dietary supplement S-adenosyl-L-methionine. Ann. Pharmacother..

[10] Stramentinoli G., Gualano M., Galli-Kienle M. (1979). Intestinal absorption of S-adenosl-L-methionine. J. Pharmacol. Exp. Ther..

[11] Noureddin M., Sander-Struckmeier S., Mato J.M. (2020). Early treatment efficacy of S-adenosylmethionine in patients with intrahepatic cholestasis: A systematic review. World J. Hepatol..

[12] Gao J., Cahill C.M., Huang X., Roffman J.L., Lamon-Fava S., Fava M., Mischoulon D., Rogers J.T. (2018). S-Adenosyl Methionine and Transmethylation Pathways in Neuropsychiatric Diseases Throughout Life. Neurotherapeutics.

[13] Ding W., Higgins D.P., Yadav D.K., Godbole A.A., Pukkila-Worley R., Walker A.K. (2018). Stress-responsive and metabolic gene regulation is altered in low S-adenosylmethionine. PLoS Genet..

[14] Mahmood N., Arakelian A., Cheishvili D., Szyf M., Rabbani S.A. (2020). S-adenosylmethionine in combination with decitabine shows enhanced anti-cancer effects in repressing breast cancer growth and metastasis. J. Cell Mol. Med..

[15] Chik F., Machnes Z., Szyf M. (2014). Synergistic anti-breast cancer effect of a combined treatment with the methyl donor S-adenosylmethionine and the DNA methylation inhibitor 5-aza-2′-deoxycytidine. Carcinogenesis.

[16] Bray N.L., Pimentel H., Melsted P., Pachter L. (2016). Near-optimal probabilistic RNA-seq quantification. Nat. Biotechnol..

[17] Ritchie M.E., Phipson B., Wu D., Hu Y., Law C.W., Shi W., Smyth G.K. (2015). Limma powers differential expression analyses for RNA-sequencing and microarray studies. Nucleic Acids Res..

[18] Sergushichev A.A., Loboda A.A., Jha A.K., Vincent E.E., Driggers E.M., Jones R.G., Pearce E.J., Artyomov M.N. (2016). GAM: A web-service for integrated transcriptional and metabolic network analysis. Nucleic Acids Res..

[19] Geistlinger L., Csaba G., Zimmer R. (2016). Bioconductor’s EnrichmentBrowser: Seamless navigation through combined results of set- & network-based enrichment analysis. BMC Bioinform..

[20] Ye C., Jiang N., Zheng J., Zhang S., Zhang J., Zhou J. (2023). Epigenetic therapy: Research progress of decitabine in the treatment of solid tumors. Biochim. Biophys. Acta Rev. Cancer.

[21] Lin S.R., Wen Y.C., Yeh H.L., Jiang K.C., Chen W.H., Mokgautsi N., Huang J., Chen W.Y., Liu Y.N. (2020). EGFR-upregulated LIFR promotes SUCLG2-dependent castration resistance and neuroendocrine differentiation of prostate cancer. Oncogene.

[22] Zhu Z.H., Yu Y.P., Zheng Z.L., Song Y., Xiang G.S., Nelson J., Michalopoulos G., Luo J.H. (2010). Integrin alpha 7 interacts with high temperature requirement A2 (HtrA2) to induce prostate cancer cell death. Am. J. Pathol..

[23] Lim J., Rodriguez R., Williams K., Silva J., Gutierrez A.G., Tyler P., Baharom F., Sun T., Lin E., Martin S. (2024). The Exonuclease TREX1 Constitutes an Innate Immune Checkpoint Limiting cGAS/STING-Mediated Antitumor Immunity. Cancer Immunol. Res..

[24] Técher H., Gopaul D., Heuzé J., Bouzalmad N., Leray B., Vernet A., Mettling C., Moreaux J., Pasero P., Lin Y.L. (2024). MRE11 and TREX1 control senescence by coordinating replication stress and interferon signaling. Nat. Commun..

[25] Miller K.N., Victorelli S.G., Salmonowicz H., Dasgupta N., Liu T., Passos J.F., Adams P.D. (2021). Cytoplasmic DNA: Sources, sensing, and role in aging and disease. Cell.

[26] Tani T., Mathsyaraja H., Campisi M., Li Z.H., Haratani K., Fahey C.G., Ota K., Mahadevan N.R., Shi Y., Saito S. (2024). TREX1 Inactivation Unleashes Cancer Cell STING-Interferon Signaling and Promotes Antitumor Immunity. Cancer Discov..

[27] Decout A., Katz J.D., Venkatraman S., Ablasser A. (2021). The cGAS-STING pathway as a therapeutic target in inflammatory diseases. Nat. Rev. Immunol..

[28] Grisanzio C., Signoretti S. (2008). p63 in prostate biology and pathology. J. Cell Biochem..

[29] Tucci P., Agostini M., Grespi F., Markert E.K., Terrinoni A., Vousden K.H., Muller P.A., Dötsch V., Kehrloesser S., Sayan B.S. (2012). Loss of p63 and its microRNA-205 target results in enhanced cell migration and metastasis in prostate cancer. Proc. Natl. Acad. Sci. USA.

[30] Lo U.G., Pong R.C., Yang D., Gandee L., Hernandez E., Dang A., Lin C.J., Santoyo J., Ma S., Sonavane R. (2019). IFNγ-Induced IFIT5 Promotes Epithelial-to-Mesenchymal Transition in Prostate Cancer via miRNA Processing. Cancer Res..

[31] Tamura K., Furihata M., Chung S.Y., Uemura M., Yoshioka H., Iiyama T., Ashida S., Nasu Y., Fujioka T., Shuin T. (2009). Stanniocalcin 2 overexpression in castration-resistant prostate cancer and aggressive prostate cancer. Cancer Sci..

