# Supplementary material for: The Simultaneous Treatment of PC-3 Cells with the DNA-Demethylating Agent Decitabine and S-Adenosylmethionine Leads to Synergistic Anticancer Effects
Source: Genes (Basel). 2024 Dec 20;15(12):1634. doi: 10.3390/genes15121634 (PMC11675482; doi:10.3390/genes15121634)
Supplement: Supplementary file 1 [file genes-15-01634-s001.zip › ZIP Kopie/Supplementary Figure 1.docx]

**Supplementary Figure 1**

STC2

**CCGACTCAGGAGAGCTC**GACACGCCGGATAGCTGCGGCCA

GCCGTGGCCATCCTGGCCCCCCGCCTCCGCCTTCCCCACT

CCCGCGTGCAGCCGGGACACGGGAAAGGAAAGCTTTGGAA

GTCAAGCGCCGGCCAAAAGATGACCTGCCCGCGTGTCTCC

TCGCCCCCTCC**CCCAGCCGTGTCACATG**

IFIT5

**GAAGCAGGGACTTAAGTTTC**CGCCTGTAATCCCAGCACCTTGG

GAGGCAGAGGCGCATCCCTTGAGATCACGACTGGGTGACACAG

TGAGACGCTATCTCAAACAAACAAACAAACAAACAAACAGTTA

CACGGGCAGCATGCGAACTCAGCGTACTCAGGGAGGCAGATAA

AAGCCGAGACCAAAGTGAAGGAGCCCAGCTGCAAATTCAACTCC

GAAAGACCCTGGACTCCGCAAACGTCTGGCCGCG**GCTCTTGAGC**

**TCTTCTATTAA**

TP63

**ACAACAGTAGAGAGGATGCCC**AGCTGGTAAGAATCGAGTGTTT

ATGAAGTTTTAGTCAATTGATGAATCTCATTGGCTAAAATCAA

GAAACGCTCCGCCTCTTTGCAAATATGTATGAAGGAGAGAAGT

GCCTAAACTTCTATGTCTGATAGCATTTGACCCTATTGCTTTT

AGCCTCCCGGCTTTATATCTATATATACACAGGTATATGTGTA

TATTTTATATAATTGTTCTCCGTTCGTTGATATCAAAGACAGT

TGAAGGAAATGAATTTTGAAACTTCACGGTGTGCCACCCTACA

GTACTGCCCTGACCCTTACATCCAGCGG**TGAGTTTGAATGTGA**

**CATAACT**
